# Respiratory Quinone Switches from Menaquinone to Polyketide Quinone during the Development Cycle in *Streptomyces* sp. Strain MNU77

**DOI:** 10.1128/spectrum.02597-22

**Published:** 2022-12-12

**Authors:** Kritee Mehdiratta, Sonam Nain, Meenakshi Sharma, Shubham Singh, Sonali Srivastava, Bhushan Dilip Dhamale, Debasisa Mohanty, Siddhesh S. Kamat, Vivek T. Natarajan, Rakesh Sharma, Rajesh S. Gokhale

**Affiliations:** a National Institute of Immunology, New Delhi, India; b Academy of Scientific and Innovative Research (AcSIR), Ghaziabad, India; c Department of Biology, Indian Institute of Science Education and Research, Pune, Maharashtra, India; d CSIR-Institute of Genomics and Integrative Biology, New Delhi, India; Indian Institute of Science Bangalore

**Keywords:** respiration, polyketide quinones, type III PKS, *Streptomyces* sp., *Streptomyces*, hypoxia

## Abstract

Type III polyketide synthases (PKSs) found across *Streptomyces* species are primarily known for synthesis of a vast repertoire of clinically and industrially relevant secondary metabolites. However, our understanding of the functional relevance of these bioactive metabolites in *Streptomyces* physiology is still limited. Recently, a role of type III PKS harboring gene cluster in producing alternate electron carrier, polyketide quinone (PkQ) was established in a related member of the *Actinobacteria*, *Mycobacteria*, highlighting the critical role these secondary metabolites play in primary cellular metabolism of the producer organism. Here, we report the developmental stage-specific transcriptional regulation of homologous type III PKS containing gene cluster in freshwater *Streptomyces* sp. strain MNU77. Gene expression analysis revealed the type III PKS gene cluster to be stringently regulated, with significant upregulation observed during the dormant sporulation stage of *Streptomyces* sp. MNU77. In contrast, the expression levels of only known electron carrier, menaquinone biosynthetic genes were interestingly found to be downregulated. Our liquid chromatography–high-resolution mass spectrometry (LC-HRMS) analysis of a metabolite extract from the *Streptomyces* sp. MNU77 spores also showed 10 times more metabolic abundance of PkQs than menaquinones. Furthermore, through heterologous complementation studies, we demonstrate that *Streptomyces* sp. MNU77 type III PKS rescues a respiratory defect of the Mycobacterium smegmatis type III PKS deletion mutant. Together, our studies reveal that freshwater *Streptomyces* sp. MNU77 robustly produces novel PkQs during the sporulation stage, suggesting utilization of PkQs as alternate electron carriers across *Actinobacteria* during dormant hypoxic conditions.

**IMPORTANCE** The complex developmental life cycle of *Streptomyces* sp. mandates efficient cellular respiratory reconfiguration for a smooth transition from aerated nutrient-rich vegetative hyphal growth to the hypoxic-dormant sporulation stage. Polyketide quinones (PkQs) have recently been identified as a class of alternate electron carriers from a related member of the *Actinobacteria*, *Mycobacteria*, that facilitates maintenance of membrane potential in oxygen-deficient niches. Our studies with the newly identified freshwater *Streptomyces* sp. strain MNU77 show conditional transcriptional upregulation and metabolic abundance of PkQs in the spore state of the *Streptomyces* life cycle. In parallel, the levels of menaquinones, the only known *Streptomyces* electron carrier, were downregulated, suggesting deployment of PkQs as universal electron carriers in low-oxygen, unfavorable conditions across the *Actinobacteria* family.

## INTRODUCTION

Type III polyketide synthases (PKSs) are a structurally and functionally simple class of PKSs that catalyze condensation of one to several molecules of extender substrate onto a starter substrate through iterative decarboxylations (1, 2). While initially thought to be present only in plants, the first bacterial type III PKS, RppA was reported from Streptomyces griseus in 1999 (3, 4). Subsequent genome projects have led to identification of type III PKS-encoding genes across *Streptomyces* sp. producing chemically diverse clinically and industrially relevant metabolic end products (5–7). Using a heterologous overexpression system of Streptomyces lividans, a new pathway for producing phenolic lipids through type III PKS has been reported for Streptomyces griseus (8). These metabolites contain a benzoquinone or a pyrone scaffold with a long alkyl chain. The benzoquinone ring is derived from an alkyl resorcinol formed by type III PKS that is modified by two downstream enzymes, a methyltransferase and a hydroxylase, both of which are present in an operon. However, the metabolite could not be isolated from the native growth conditions of *Streptomyces*, nor could their physiological role be deciphered (8). Recent characterization of alkyl benzoquinones biosynthesized from a similar type III PKS operon from Mycobacterium smegmatis, named polyketide quinones, elucidated the functional role of these metabolites as alternate electron carriers during respiration under hypoxic growth conditions (9). Their production and role in *Streptomyces* biology, however, remain to be elucidated.

*Streptomyces*, a soil bacterium, is ubiquitous in nature. Its ability to colonize the soil is greatly facilitated by growth as a vegetative hyphal mass which can differentiate into spores that assist in spread and persistence (10, 11). The spores are a semi dormant stage in the life cycle that can survive in soil for long periods of nutrient and oxygen deficiency (12–14). However, the respiratory network that enables *Streptomyces* spores to sustain the dramatic effects of hypoxia and maintain proton motive force is not completely understood. *Streptomyces* colonies are thus multicellular, differentiated organisms exhibiting temporal and spatial control of gene expression, morphogenesis, metabolism, and the flux of metabolites.

Biogeographically, *Streptomyces* species have a wide distribution, since they can be found in diverse habitats such as polar territories, deserts, highlands, wetlands, and marine sediments (15–18). As a result of their clinical significance, multiple *Streptomyces* strains are isolated every year. Most of them represent already characterized species displaying similar or even identical genomic features, though their bioactive potential may vary vastly (19, 20). In addition to the core primary metabolites, many *Streptomyces* strains have an accessory secondary metabolite chemical arsenal which has not been completely studied (21). Moreover, diverse experiments have demonstrated that the different developmental stages of *Streptomyces* may activate cryptic biosynthetic pathways, producing uncommon or unknown metabolites (22–24).

In this study, we report a new strain of freshwater *Streptomyces*, *Streptomyces* sp. MNU77, isolated from the Indus River, Leh, India. Genome sequencing and phylogenetic analysis revealed *Streptomyces* sp. MNU77 to be a distinct member of the genus *Streptomyces*. Computational analysis further showed the genome to be endowed with a variety of biosynthetic gene clusters involved in the production of diverse secondary metabolites such as polyketides, pyrones, ribosomally synthesized and posttranslationally modified peptides, and nonribosomal peptides. Interestingly, only one type III PKS could be mapped to the genome of *Streptomyces* sp. MNU77, which occurred in an operonic arrangement similar to that of M. smegmatis polyketide quinone (PkQ)-producing type III PKS gene clusters. Exploiting the hallmark complex developmental cycle of *Streptomyces*, we identified and characterized the production of PkQs specifically from the spore stage of *Streptomyces* sp. MNU77, suggesting their role in maintenance of respiratory homeostasis during the dormant sporulation growth phase.

## RESULTS

### Genomic and phylogenetic features of *Streptomyces* sp. MNU77.

*Streptomyces* sp. strain *MNU77* was isolated from the freshwater Indus River in Leh, India. Interestingly, only 8 *Streptomyces* species have been reported to date from freshwater ecosystems (25–27). The genome of *Streptomyces* sp. MNU77 was sequenced using PacBIO technology and assembled using Hierarchical Genome Assembly Process (HGAP) v.2.0. The draft genome of *Streptomyces* sp. MNU77, consisting of 8,868,350 bp, was assembled into 4 contigs with a 131× genome coverage and an *N*_50_ value of 8,219,841 bp. The genome of *Streptomyces* sp. MNU77 has a high G+C content of 71.7%. The genome annotation for strain MNU77 includes a total of 7,729 coding sequences (CDSs) and 67 tRNA genes (see Fig. S1a in the supplemental material). In contrast to soil-dwelling *Streptomyces* species, only 2 rRNA genes are present in the *Streptomyces* sp. MNU77 genome. The 16S rRNA gene of *Streptomyces* sp. MNU77 interestingly showed 100% sequence identity with the soil-dwelling, obligate aerobe Streptomyces pratensis ch24 and 99.4% identity with Streptomyces annulatus, also found in soil. Phylogenetic analysis with the closest strains identified from EzBioCloud similarly revealed *Streptomyces* sp. MNU77 to be closely related to Streptomyces pratensis ch24 (Fig. S1b).

### Transcriptional regulation of menaquinone biosynthetic genes during the *Streptomyces* sp. MNU77 life cycle.

**FIG 1:**
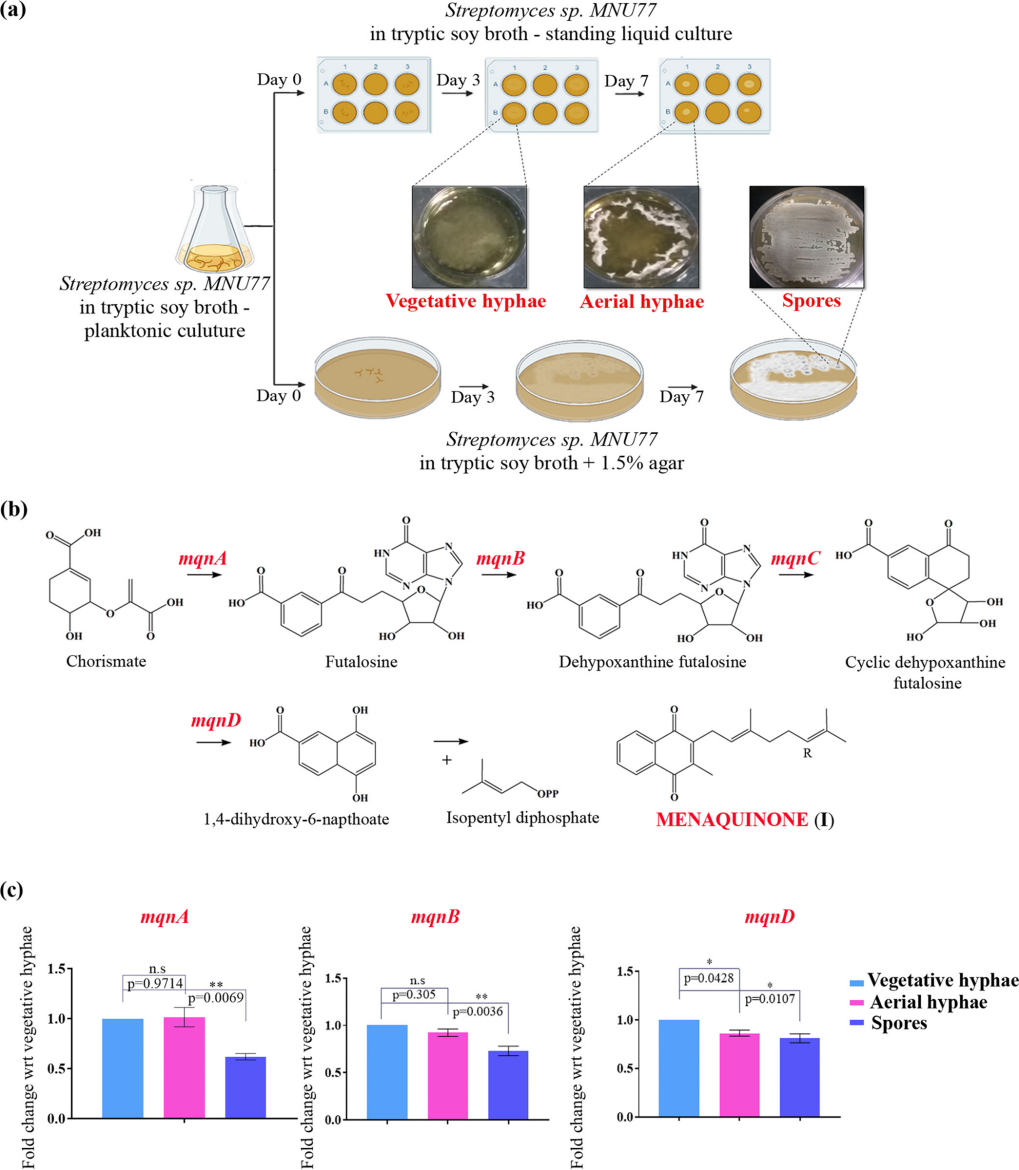
Conditional regulation of menaquinone biosynthesis in *Streptomyces* sp. MNU77. (a) Experimental setup with morphological comparison of *Streptomyces* sp. MNU77 vegetative hyphae, aerial hyphae, and spores. (b and c) Biosynthetic pathway and qRT PCR-based gene expression analysis of menaquinone pathway in vegetative hyphae, aerial hyphae, and spores of *Streptomyces* sp. MNU77. *P* values were determined by one-way analysis of variance (ANOVA) with Dunnett posttests with respect to (wrt) to vegetative hyphae and are shown above each data point. Data represent the mean ± the standard error of the mean (SEM) (*n* = 3).

The three distinct developmental stages of *Streptomyces*—vegetative hyphae, aerial hyphae, and spores—experience various levels of nutrient exhaustion and environmental stress (28). In order to enrich all 3 phases separately to understand the underlying mechanism of biosynthesis of quinone metabolites, we employed a combination of a standing liquid model and a solid-phase agar model of growth for 7 days. Submerged vegetative hyphae attached to the plate surface could be observed by day 3 in standing liquid culture and then migrated to the liquid-air interface to form aerial hyphal structures by day 7, which were collected. The gray pigmented *Streptomyces* sp. MNU77 spores were harvested from a solid-phase medium of tryptic soy broth supplemented with 1.5% agar (Fig. 1a). To understand the underlying respiratory quinone machinery enabling efficient phase-dependent adaptation to the environment, we performed quantitative reverse transcriptase PCR (qRT-PCR)-based gene expression analysis of the only known *Streptomyces* electron carrier, the menaquinone biosynthetic pathway, in vegetative hyphae, aerial hyphae, and spores of *Streptomyces* sp. MNU77. *Streptomyces* utilizes a futalosine-based alternate biosynthetic pathway, employing genes *mqnA, mqnB, mqnC* and *mqnD* for biosynthesis of menaquinone (29, 30) (Fig. 1b). The gene expression levels of *mqnA* and *mqnB* were found not to differ much between aerial and vegetative hyphae, while *mqnD* was found to be 0.2 log-fold downregulated in the aerial hyphae. The expression levels of *mqnA*, *mqnB* and *mqnD* genes, on the other hand, were found to be significantly downregulated in *Streptomyces* sp. MNU77 spores compared to the vegetative hyphal stage (Fig. 1c). These results thus demonstrate phase-dependent expression of the menaquinone biosynthetic pathway, which is suggestive of a decrease in menaquinone production.

### The *Streptomyces* sp. MNU77 genome possesses multiple secondary metabolite biosynthetic gene clusters.

**FIG 2:**
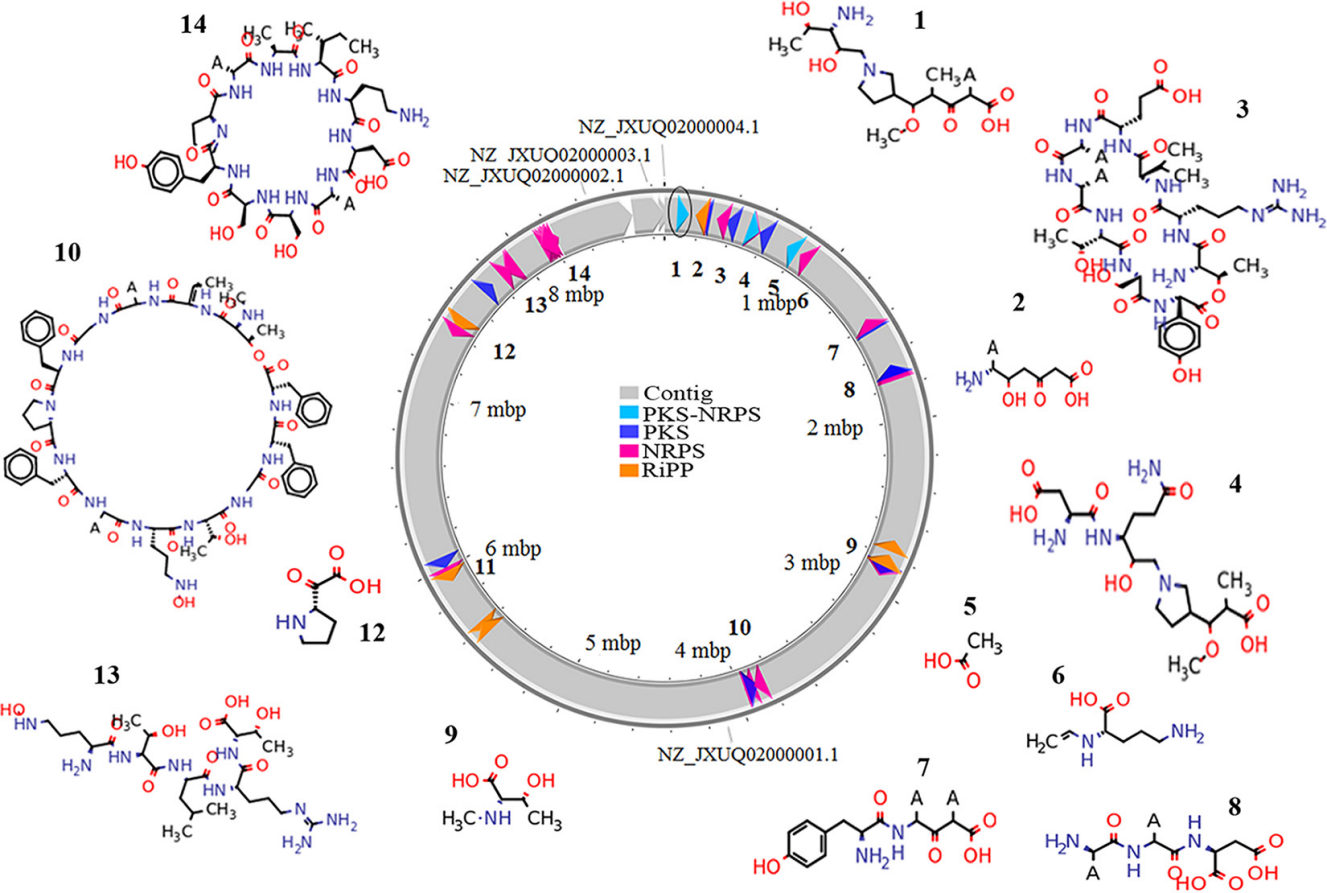
The *Streptomyces* sp. MNU77 genome possesses multiple biosynthetic clusters for secondary metabolite production. Genomic coordinates for putative secondary metabolites producing biosynthetic clusters in the genome of *Streptomyces* sp. MNU77 are shown.

Recently a type III PKS operon characterized from M. smegmatis was shown to produce a novel metabolite, polyketide quinone (PkQ), which facilitates respiration under low-oxygen conditions (9). We thus performed computational analysis of the *Streptomyces* sp. MNU77 genome using antiSMASH v.5, PRISM, SBSPKS v.3, and RiPPMiner-Genome to search for such bioactive natural product biosynthetic gene clusters such as polyketide synthases (PKSs), nonribosomal peptide synthases (NRPSs), and ribosomally synthesized and posttranslationally modified peptides (RiPPs). The analysis revealed rich bioactive potential with a total of 35 putative secondary metabolite-producing gene clusters identified from the genome of freshwater *Streptomyces* sp. MNU77. A total of 22 clusters corresponded to PKSs, NRPSs, PKS-NRPS hybrids, and RiPPs. Out of these 22 PKSs, NRPSs, PKS-NRPSs, and RiPP biosynthetic gene cluster (BGC), the chemical structure of the biosynthetic product could be predicted using software such as SBSPKS v.3 and RiPPMiner-Genome for 14 BGCs which showed significant gene cluster-level similarity to known BGCs (Fig. 2). However, it must be noted that BGC similarity is measured as the percentage of genes in a known BGC which share significant sequence similarity with genes in the predicted BGC. Hence, the chemical structure of secondary metabolites predicted by these software programs is likely to have only scaffold-level similarity with known molecules such as nucleocidin, streptobactin, AmfS, cycloheximide, warkmycin, RP-1776 etc. Interestingly, out of the 35 potential biosynthetic gene clusters, only 15 showed significant gene cluster similarity (>50%) with known biosynthetic gene clusters, whereas 16 clusters showed weak homology (Table 1). Notably, for 4 clusters, no homologous gene clusters could be identified, and they hence represent orphan BGCs (Table 1). The analysis thus suggests *Streptomyces* sp. MNU77 to have strong bioactive potential for production of novel secondary metabolites.

**TABLE 1:** Putative secondary metabolites producing biosynthetic clusters of *Streptomyces* sp. MNU77 with homology to the most similar known biosynthetic clusters as predicted by antiSMASH

Region (contig.cluster)	Start (bp)	End (bp)	Size (bp)	Most similar known biosynthetic cluster	Similarity (%)
Terpenes					
1.1	921518	947729	26,211	Hopene	69
1.11	1332855	1353079	20,224	2-methylisoborneol	100
1.15	2325366	2346259	20,893		
1.26	7071455	7090526	19,071	Steffimycin-D	19
1.31	8032817	8055030	22,213	Geosmin	100
3.1	55537	81150	25,613	Isorenieratene	100
NRPS					
1.9	841017	890506	49,489	Nucleocidin	47
1.27	7396863	7441615	44,752	Borrelidin	9
1.3	7844052	7934657	90,605	Streptobactin	94
1.33	8133437	8219841	86,404	Marformycin A	16
Siderophores					
1.14	1914109	1927122	13,013	Ficellomycin	3
1.22	5495770	5507548	11,778	Desferrioxamin B	80
PKS					
1.6 (type 1)	575859	621549	45,690	C-1027	17
1.29 (type 3)	7712481	7753599	41,118	Herboxidiene	6
Peptides					
1.16 (Lanthipeptide-class-3)	2723192	2744995	21,803	AmfS	100
1.18 (thiopeptide, LAP)	2809899	2837740	27,841	Frigocyclinone	17
1.23 (lanthipeptide-class-2,3)	5575438	5606294	30,856	-	
1.28 (thiopeptide, LAP)	7459145	7489335		Lividomycin	6
Butyrolactones					
1.32	8086413	8097357	10,944	Coelimycin P1	8
Hybrids					
1.1 (T1PKS, NRPS)	81191	133507	52,316	Kanamycin	2
1.2 (NRPS-like, lanthipeptide-class-4, transAT-PKS)	196649	268643	71,994	Cycloheximide	88
1.3 (NRPS, T3PKS)	294505	417357	122,852	Lipopeptide 8D1-1	8
1.5 (NRPS, T1PKS)	504173	556650	52,477	Dutomycin	4
1.8 (NRPS, T1PKS)	751367	800407	49,040	SGR PTMs	100
1.13 (T2PKS, oligosaccharide, NRPS)	1662722	1743622	80,900	Warkmycin CS1	97
1.19 (T1PKS, NRPS)	2853943	2907212	53,269	Enduracidin	6
1.20 (ectoine, butyrolactone, ladderane, arylpolyene, NRPS, T1PKS)	3812110	3951038	138,928	RP-1776	95
1.23 (lanthipeptide-class-3, lanthipeptide-class-2)	5575438	5606294	30,856		
1.24 (lanthipeptide-class-2, NRPS, RRE-containing)	5932721	5990452	57,731	CDA1b	10
Others					
1.4 (melanin)	451858	459602	7,744	Melanin	100
1.7 (RiPP-like)	722678	732935	10,257	Tetronasin	3
1.12 (RiPP-like)	1563226	1574617	11,391		
1.17 (melanin)	2784901	2795323	10,422	Melanin	100
1.21 (ectoine)	4708402	4717367	8,965	Ectoine	75
1.25 (ectoine)	6625052	6635450	10,398	Ectoine	100

### Transcriptional regulation of a type III PKS operon in *Streptomyces* sp. MNU77.

**FIG 3:**
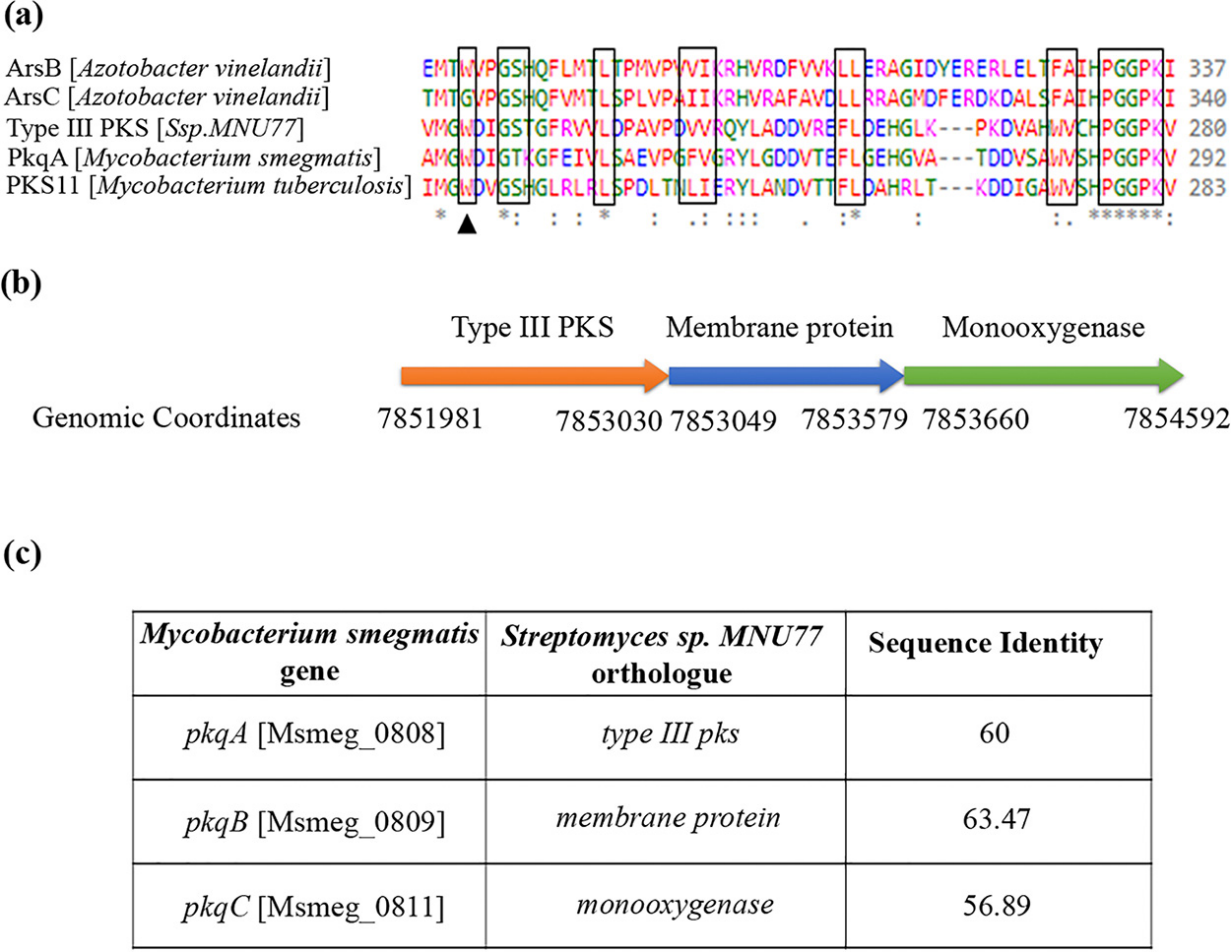
Bioinformatic analysis of *Streptomyces* sp. MNU77 type III PKS biosynthetic gene cluster. (a) Sequence alignment of type III PKS proteins from *Azotobacter* spp., M. smegmatis, M. tuberculosis, and *Streptomyces* sp. MNU77 shows conservation of critical catalytic residue tryptophan at position 281. (b) Genomic coordinates of type III PKS operon in *Streptomyces* sp. MNU77. (c) Sequence homology of *Streptomyces* sp. MNU77 putative type III PKS biosynthetic gene cluster with M. smegmatis polyketide quinone biosynthesis operon.

**FIG 4:**
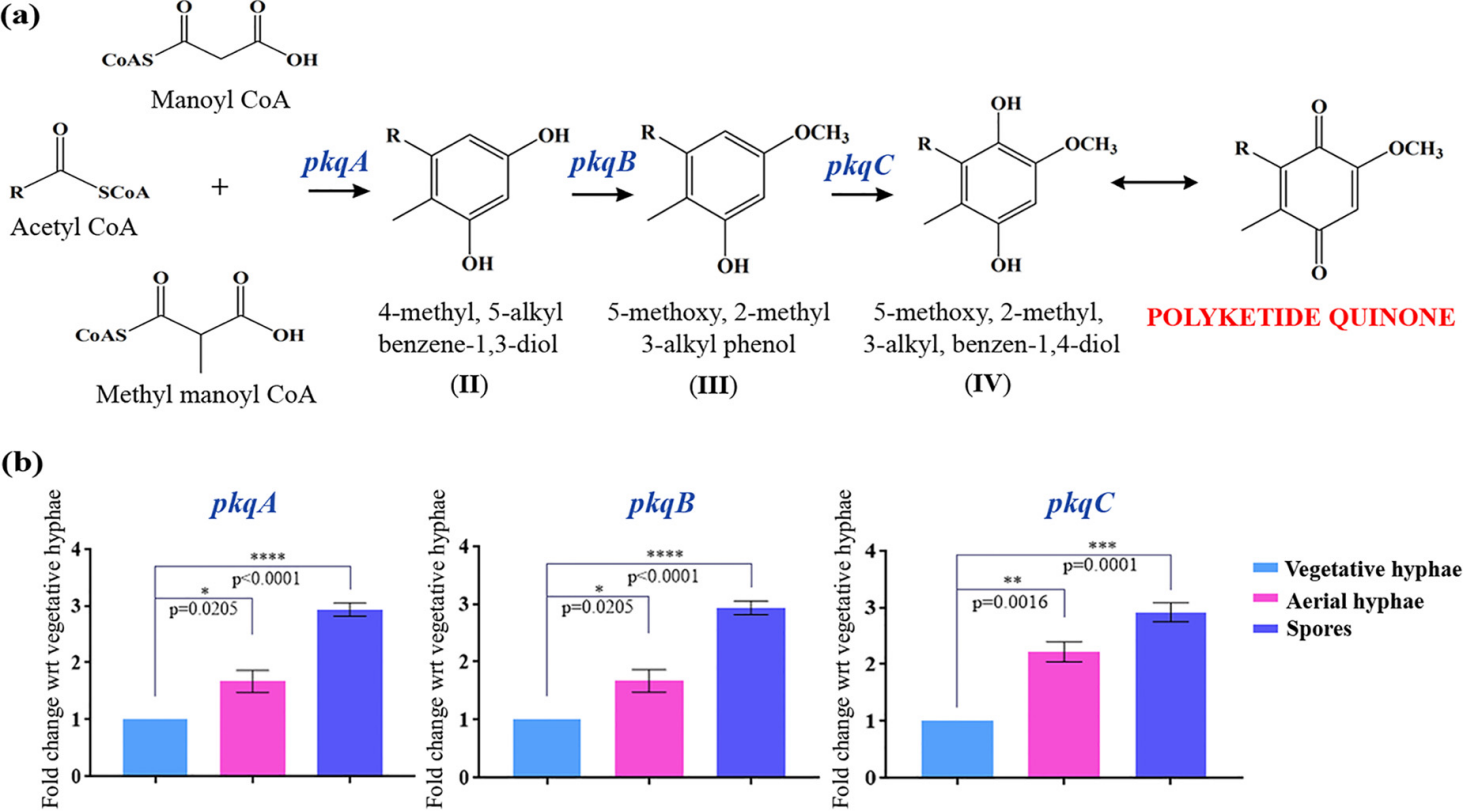
Conditional regulation of polyketide quinone biosynthetic gene cluster in *Streptomyces* sp. MNU77. (a) Pathway for biosynthesis of polyketide quinones using acetyl-coenzyme A (CoA), malonyl CoA, and methylmalonyl CoA as starter and extender substrates respectively. (b) qRT PCR-based gene expression analysis of polyketide quinone biosynthetic genes in vegetative hyphae, aerial hyphae, and spores of *Streptomyces* sp. MNU77. *P* values were determined by one-way ANOVA with Dunnett posttests with respect to (wrt) to vegetative hyphae and are shown above each data point. Data represent the mean ± SEM (*n* = 3).

The bioinformatics studies interestingly revealed the presence of only one type III PKS gene in the *Streptomyces* sp. MNU77 genome. We then performed multiple-sequence alignment of *Streptomyces* sp. MNU77 with other characterized type III PKSs from *Azotobacter* sp. ArsB and ArsC, M. smegmatis PkqA, and Mycobacterium tuberculosis PKS11. In an earlier study with an *Azotobacter* sp., type III PKSs ArsB and ArsC were shown to produce resorcinol and pyrone, respectively (31, 32). This difference was partially attributed to the presence of tryptophan residue at position 281 of ArsB, which is glycine at ArsC (32). Like ArsB and M. smegmatis *pkqA*, multiple-sequence alignment results demonstrated the *Streptomyces* sp. MNU77 type III PKS to also contain tryptophan at position 281 (Fig. 3a). Furthermore, the genomic loci harboring putative type III PKS from 7,851,981 bp to 7,853,030 bp was found to be present in an operonic arrangement with two downstream genes that showed significant similarity with methyltransferase and oxidoreductase of the M. smegmatis type III PKS operon (*pkqA* to *pkqC*) (Fig. 3b). Interestingly, this 3-gene PkQ biosynthetic machinery was previously reported to be conserved across different *Actinomycetes* species, including some members of the *Streptomyces* genus (9). The analysis thus suggests *Streptomyces* sp. MNU77 type III PKS to likely produce resorcinol as an end product. The two downstream genes, methyltransferase and oxidoreductase would act on the resorcinol backbone formed by type III PKS to produce PkQ (Fig. 4a). In order to investigate the PkQ biosynthetic machinery, we performed comparative transcriptional analysis of the *Streptomyces* sp. MNU77 type III PKS three-gene cluster—type III PKS, methyltransferase, and oxidoreductase—under the different growth stages of the *Streptomyces* development cycle. While a log fold induction was observed for PkQ biosynthetic genes in aerial hyphae, the entire gene cluster was found to be 2- to 3-log fold upregulated in spores of *Streptomyces* sp. MNU77 compared to vegetative hyphae (Fig. 4b). The life cycle-dependent transcriptional induction of the type III PKS operon thus demonstrates stringently regulated conditional relevance of the product produced. It is also noteworthy that the increased expression of PkQ biosynthetic machinery is anticorrelated with the menaquinone biosynthetic pathway in the *Streptomyces* life cycle, suggestive of a quinone compensatory mechanism previously observed for M. smegmatis.

### *Streptomyces* sp. *MNU77* spores produce polyketide quinones.

**FIG 5:**
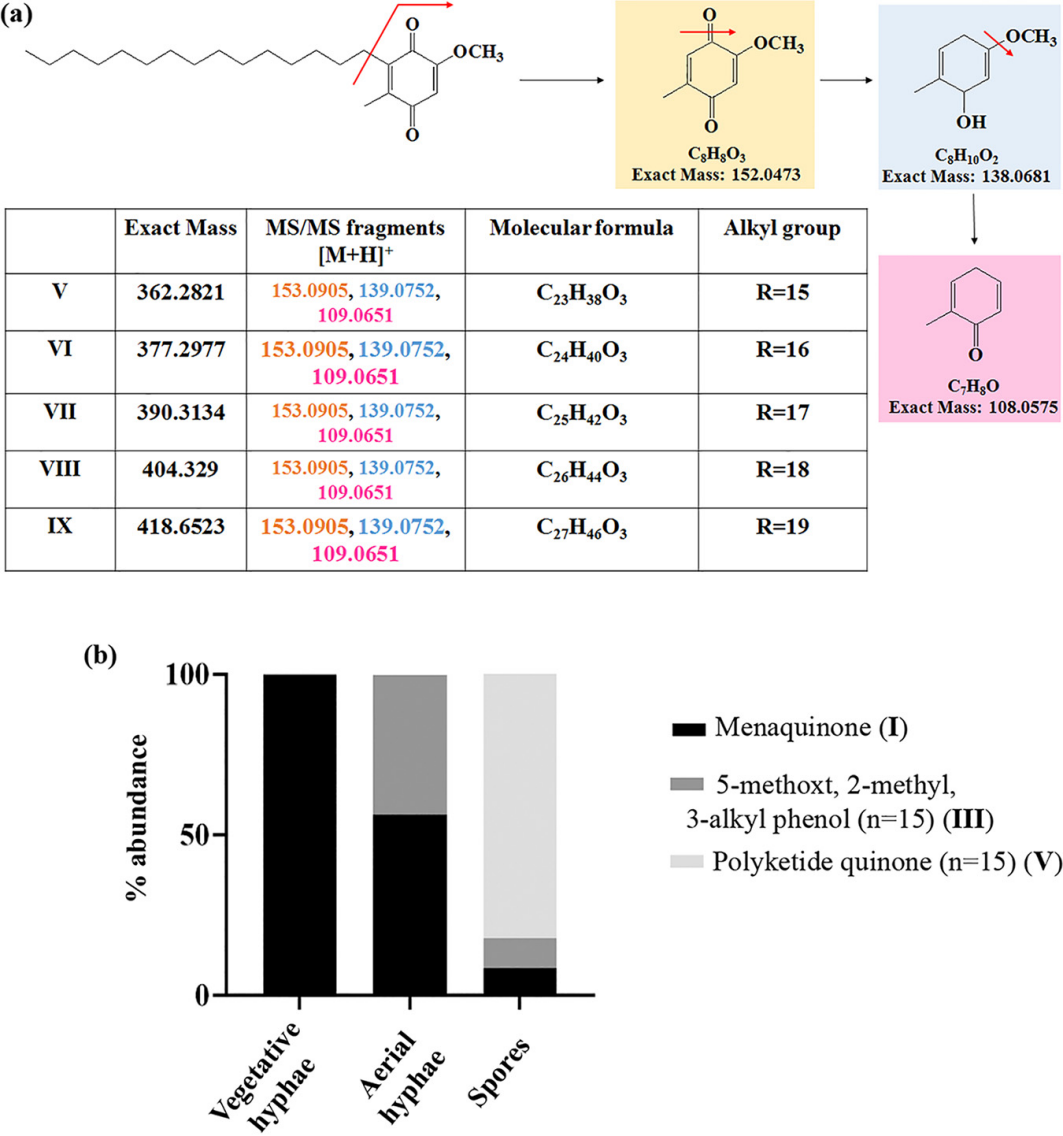
Identification and characterization of polyketide quinones from *Streptomyces* sp. MNU77. (a) Common MS/MS fragments identified in the positive ion mode for all the identified PkQ masses (V to IX). The predicted fragments are color-coded along with their masses in the table. (b) Relative abundance of menaquinone (I), intermediate of PkQ biosynthetic pathway (III), and PkQ (V) metabolites in vegetative hyphae, aerial hyphae, and spores of *Streptomyces* sp. MNU77.

To ascertain whether this enhanced transcriptional induction of the type III PKS pathway translates to production of PkQs, we performed high-resolution liquid chromatograph-mass spectrometry (LC-MS) analysis of metabolites isolated from vegetative hyphae, aerial hyphae, and spores of *Streptomyces* sp. MNU77. Ultra-high-pressure liquid chromatography (UHPLC) analysis of metabolite extract from vegetative hyphal cultures showed no peaks with retention time similar to those of chemically synthesized PkQ that had retention time of 22 min (Fig. S2a). In contrast, UHPLC analysis of metabolite extract from spores showed several peaks with retention time ranging from 17 min to 25 min (Fig. S2b). High-resolution LC-MS analysis of these collected peaks identified them as molecular ion peaks with *m/z* [M+H]^+^ 363.2894 (metabolite V), 377.3050 (metabolite VI), 391.3134 (metabolite VII), 405.3363 (metabolite VIII) and 419.3512 (metabolite IX) Da, all of which showed common fragmentation patterns in tandem MS/MS producing [M+H]^+^ ions of 153.0905, 139.0752, and 109.0651 (Fig. 5a, Fig. S2c). These signature fragmentation peaks are identical to those of the chemically synthesized PkQs. The difference of 14 Da across the five identified masses corresponds to “-CH_2–_” unit differences in the alkyl chain. Additionally, the three metabolic intermediates in the polyketide quinone biosynthesis pathway—4-methyl, 5-alkyl, benzene-1,4, diol (metabolite II); 5-methoxy, 2-methyl, 3-alkyl phenol (metabolite III), and 5-methoxy, 2-methyl, 3-alkyl benzene-1,4,diol (metabolite IV)—of varying chain lengths could also be detected from *Streptomyces* sp. MNU77 spores (Table 2). The findings thus provided conclusive chemical identification of polyketide quinones from *Streptomyces* sp. MNU77 spores of alkyl chain lengths varying from C_14_ to C_19_. Next, we investigated into the relative abundance of menaquinone and PkQ across the three developmental stages by liquid chromatography–high-resolution mass spectrometry (LC-HRMS) analysis. In vegetative hyphae, menaquinone (metabolite I) for *m/z* [M+H]^+^ 787.6315 could be detected robustly, but no traces of PkQ or its intermediates could be observed. On the other hand, while PkQs could not be detected in the LC-HRMS analysis of metabolites isolated from aerial hyphae of *Streptomyces* sp. MNU77, intermediates of the PkQ biosynthetic pathway (metabolite III) could be observed along with menaquinone (metabolite I) accounting for approximately 40% abundance compared to menaquinone. Interestingly, while menaquinone (metabolite I) could also be detected in the *Streptomyces* sp. MNU77 spores, the relative abundance of menaquinone was found to be a log fold lower than that of PkQ (metabolite V) in *Streptomyces* sp. MNU77 spores (Fig. 5b).

**TABLE 2:** List of intermediate metabolites of the polyketide quinone biosynthetic pathway detected in the spores of *Streptomyces* sp. MNU77

IUPAC name	[M+H]^+^
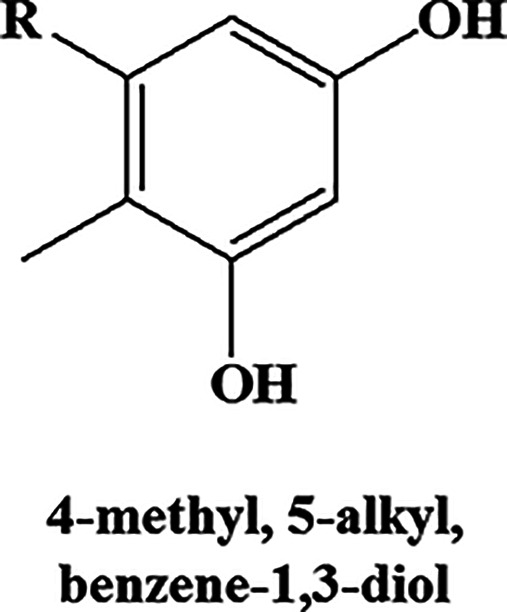	
4-Methyl-5-(12′-methyltridecyl)-benzene-1,3-diol	321.2788
4-Methyl-5-(13′-methyltetradecyl)-benzene-1,3-diol	335.2945
4-Methyl-5-(14′-methylpentadecyl)-benzene-1,3-diol	349.3101
4-Methyl-5-pentadecyl-benzene-1,3-diol	335.2945
4-Methyl-5-hexadecyl-benzene-1,3-diol	363.3258
4-Methyl-5-heptadecyl-benzene-1,3-diol	377.3414
4-Methyl-5-octadecyl-benzene-1,3-diol	391.3571
4-Methyl-5-nonadecyl-benzene-1,3-diol	405.3727
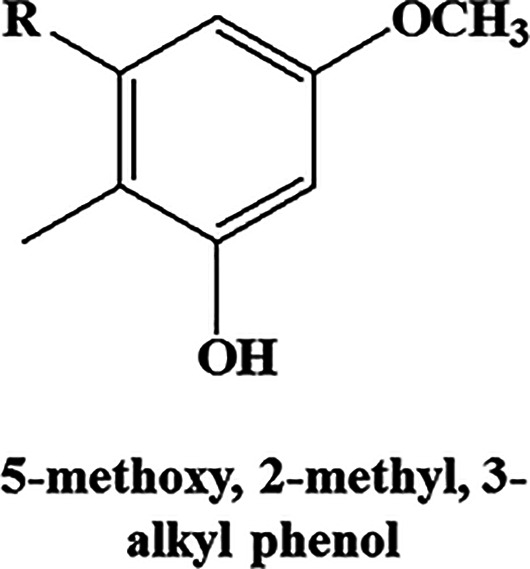	
5-Methoxy-2-methyl-3-(12'-methyltridecyl)-phenol	335.2945
5-Methoxy-2-methyl-3-(13′-methyltetradecyl)-phenol	349.3101
5-Methoxy-2-methyl-3-(13′-methylpentadecyl)-phenol	363.3258
5-Methoxy-2-methyl-3-pentadecyl-phenol	349.3101
5-Methoxy-2-methyl-3-hexadecyl-phenol	363.3258
5-Methoxy-2-methyl-3-heptadecyl-phenol	377.3414
5-Methoxy-2-methyl-3-octadecyl-phenol	391.3571
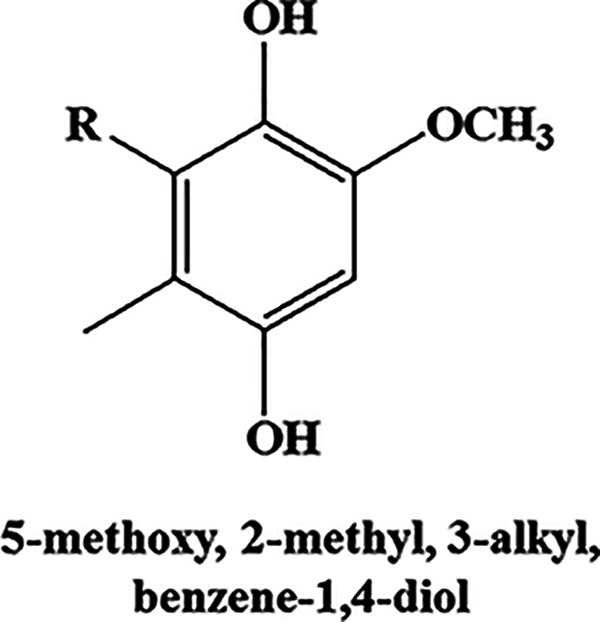	
5-Methoxy-2-methyl-3-(13′-methyltetradecyl)-benzne-1,4-diol	365.3050
5-Methoxy-2-methyl-3-(13′-methylpentadecyl)-benzene-1,4-diol	379.3207
5-Methoxy-2-methyl-3-pentadecyl-benzene-1,4-diol	365.3050
5-Methoxy-2-methyl-3-hexadecyl-benzene-1,4-diol	379.3207
5-Methoxy-2-methyl-3-heptadecyl-benzene-1,4-diol	393.3363
5-Methoxy-2-methyl-3-octadecyl-benzene-1,4-diol	407.3520
5-Methoxy-2-methyl-3-nonadecyl-benzene-1,4-diol	421.3676

### *Streptomyces* sp. *MNU77* type III PKS rescues the Mycobacterium smegmatis Δ*pkqA* biofilm defect.

**FIG 6:**
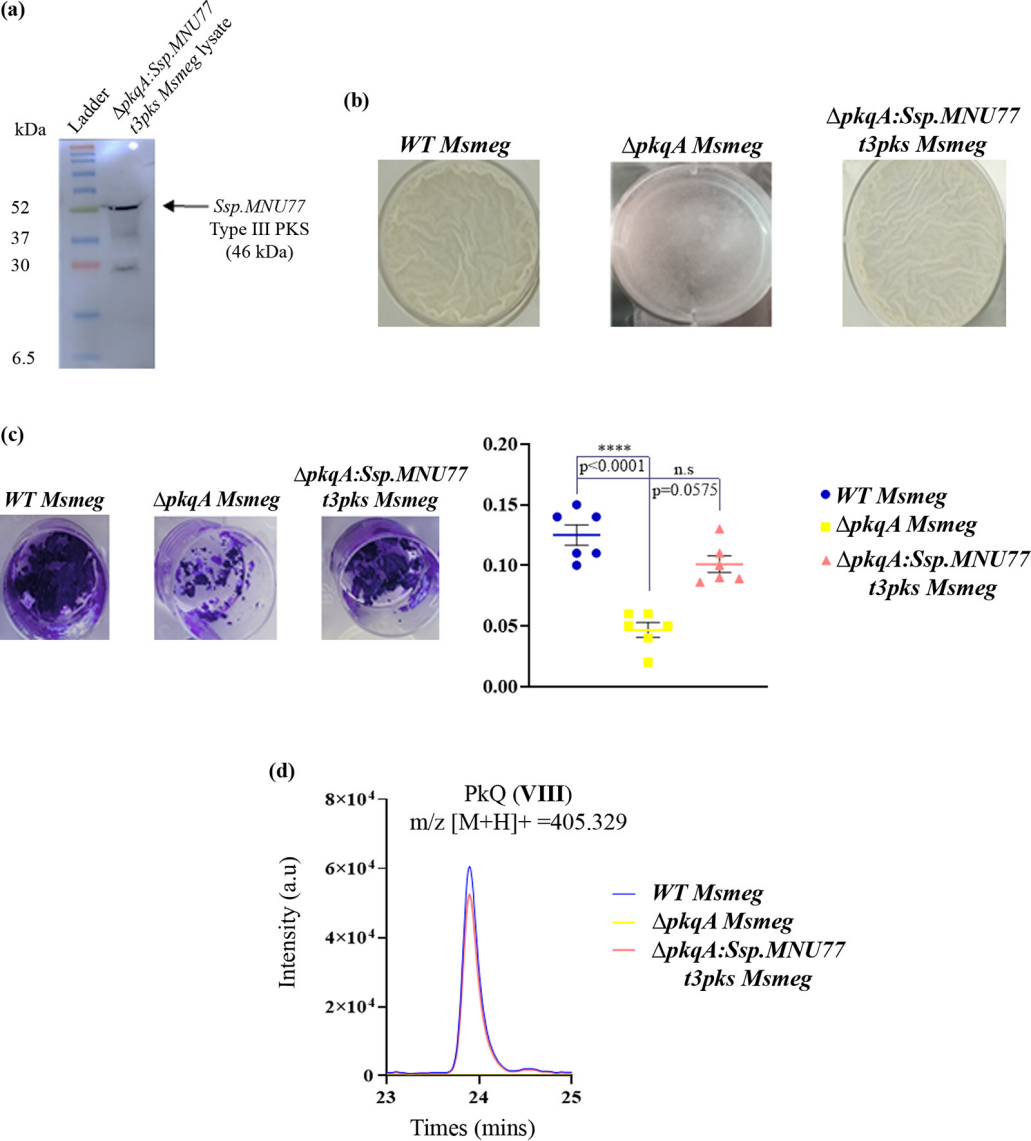
Type III PKS from *Streptomyces* sp. MNU77 rescues Δ*pkqA* Mycobacterium smegmatis. (a) Western blot of the Δ*pkqA* M. smegmatis strain expressing 3× FLAG-*Streptomyces* sp. MNU77 t3pks-His tagged protein with anti-FLAG antibody. The band of interest is marked with an arrow. (b) Biofilm formed by WT, Δ*pkqA*, and Δ*pkqA*:*Ssp.MNU77t3pks* M. smegmatis strains. Complementation of Δ*pkqA* M. smegmatis with type III PKS from *Streptomyces* sp. MNU77 rescues its biofilm formation ability. (c) Biofilm biomass for the three strains was stained with crystal violet stain and quantified by measuring the absorbance at 595 nm. *P* values were determined by one-way ANOVA with Dunnett posttests relative to WT M. smegmatis and are shown above each data point. Data represents the mean ± SEM (*n* = 6). (d) Comparative EIC of polyketide quinone VIII of *m/z* for (M+H)+ 405.329 from biofilm extracts of WT, Δ*pkqA*, Δ*pkqA*:*Ssp.MNU77t3pks* M. smegmatis strains.

We next validated the role of *Streptomyces* sp. MNU77 type III PKS by heterologous expression of the *Streptomyces* sp. MNU77*7* type III PKS protein in an M. smegmatis strain where the sole type III PKS gene, *pkqA*, is deleted. In the absence of PkQ biosynthetic machinery, the M. smegmatis strain carrying Δ*pkqA* has been shown to have impaired survival under oxygen-limiting conditions, forming fragile biofilm devoid of reticular architecture (9). The *Streptomyces* sp. MNU77 type III PKS gene was cloned in mycobacterial expression vector pST-Hi with N-terminal 3× FLAG tag and C terminal His tag and was expressed in Δ*pkqA* M. smegmatis cells. The expression was then confirmed by Western blot analysis of Δ*pkqA* M. smegmatis plus *Streptomyces* sp. MNU77 type III PKS cells with α-FLAG antibody. Indeed, a strong band correlating to 3× FLAG-*Streptomyces* sp. MNU77 type III PKS-His could be observed, confirming generation of *Streptomyces* sp. MNU77 type III PKS-expressing M. smegmatis, referred to as the Δ*pkqA*:*Ssp.MNU77t3pks* M. smegmatis strain (Fig. 6a). We then investigated the biofilm-forming ability of the Δ*pkqA*:*Ssp.MNU77t3pks* M. smegmatis strain compared to the wild type (WT) and Δ*pkqA* M. smegmatis strains. Indeed, complementation of Δ*pkqA* M. smegmatis with *Streptomyces* sp. MNU77 type III PKS rescued the defect, and robust thick biofilm with reticulations comparable to those of WT M. smegmatis biofilm could be observed (Fig. 6b), which was then quantitated by crystal violet staining and measurement of absorbance at 595 nm (Fig. 6c). In order to estimate the level of PkQ formed upon *Streptomyces* sp. MNU77 type III PKS complementation, we performed LC-HRMS analysis of the metabolite extracted from the biofilm of the three M. smegmatis strains—WT, Δ*pkqA*, and Δ*pkqA*:*Ssp.MNU77t3pks*. While no mass peak corresponding to PkQ could be detected from metabolites extracted from Δ*pkqA* M. smegmatis biofilm, comparable levels of PkQ metabolites could be detected in the WT and Δ*pkqA*:*Ssp.MNU77t3pks* M. smegmatis strains, demonstrating functional complementation by *Streptomyces* sp. MNU77 type III PKS (Fig. 6d).

## DISCUSSION

Efficient respiration culminating in generation of proton motive force (PMF) is required by all bacteria to survive and remain viable under replicating as well as nonreplicating conditions. Electron transfer by redox-active, lipophilic small molecules, quinones, between different respiratory complexes facilitates generation of this required PMF (33, 34). It is a well-established fact that Gram-negative bacteria such as Escherichia coli employ different respiratory quinones—ubiquinone and menaquinone—based on environmental oxygen availability (35–37). However, until recently, only one class of electron carrier, menaquinone, was known in Gram-positive bacteria. Our group recently reported polyketide quinones (PkQ), biosynthesized using an unconventional type III PKS machinery, as an alternate class of electron carriers (9). So far, these molecules have been isolated only from *Mycobacteria* (9), and it is not clear whether they could function as alternate respiratory quinones across other Gram-positive bacteria. In this study, we isolated and characterized this new class of respiratory quinones in *Streptomyces* sp. MNU77. Moreover, exploiting the distinct developmental life cycle of *Streptomyces* spp., we further demonstrate preference of PkQ species in the oxygen-limited sporulation stage.

In the *Streptomyces* life cycle, the spore represents the dormant phase that can efficiently survive long periods of hypoxic or even anaerobic nutritionally limited environmental conditions (12, 38). However, little is known about the cellular respiratory machinery that maintains viability of *Streptomyces* spores under such conditions of oxygen limitation. Many of the insights for the bacterium’s respiration capabilities come from the genome sequence of the model species Streptomyces coelicolor A3. Although classified as an obligate aerobe, the genome of *Streptomyces* spp. harbor three operons encoding respiratory nitrate reductases (Nar) (39, 40). Under oxygen-limitation conditions, many bacteria employ nitrate and fumarate as alternate terminal electron acceptors (41). Indeed, coping with hypoxia appears to be an important physiological challenge during the *Streptomyces* life cycle, as in addition to Nar enzymes, the *Streptomyces* genome sequence analysis revealed the bacterium to also carry respiratory cytochrome *bd* oxidase that has high-affinity for oxygen (42). Within the genome of S. coelicolor, three type III PKS genes were identified, with sco7671 type III PKS present in an operonic arrangement similar to that of the M. smegmatis *pkqA* gene cluster (43). Despite *in vitro* biochemical characterization of sco7671, the final metabolic product produced by this gene cluster could not be identified (44). Genomic investigations into our newly reported *Streptomyces* species, *Streptomyces* sp. MNU77, interestingly revealed only one type III PKS to be present. Much like sco7671, *Streptomyces* sp. MNU77 type III PKS also showed considerable homology with M. smegmatis *pkqA* and is present in an operonic arrangement with two downstream genes, demonstrating the polyketide quinone biosynthesis machinery to be conserved across *Streptomyces* species. These findings thus suggest *Streptomyces* to be equipped with adaptable metabolic and respiration machinery that confers bacteria flexibility in energy production depending on environmental conditions.

Conditional upregulation of the type III PKS containing three gene clusters and consequent identification of PkQ from *Streptomyces* sp. MNU77 specifically in the spores indicates primary metabolic functions of these secondary metabolites during specialized growth conditions and environments. The conglomeration of cells in mycobacterial biofilms is known to set in a gradient of oxygen concentration leading to production of PkQs for efficient survival and adaptation of M. smegmatis in the hypoxic biofilm environment. In the absence of PkQs, M. smegmatis with the impaired respiration machinery is unable to form biofilms (9). Indeed, complementation with *Streptomyces* sp. MNU77 type III PKS rescued this functional respiration defect of M. smegmatis Δ*pkqA*, highlighting the critical role PkQ plays for maintenance of viability under oxygen-limitation conditions. Our studies thus establish PkQ as an alternate electron carrier to menaquinone under oxygen-limitation conditions across the *Actinobacteria* family. Additionally, the differential metabolic abundance of menaquinone, intermediates of the PkQ biosynthetic pathway, and PkQ across the three stages of vegetative hyphae, aerial hyphae, and spores of *Streptomyces* sp. MNU77 suggests dynamic remodeling of the transcriptional and metabolic landscape during the developmental cycle. Upregulation of PkQ biosynthetic genes along with detection of PkQ intermediates in the aerial hyphal stage could indicate the PkQ biosynthetic machinery to be poised for progression into the next developmental stage, spores. Investigating the regulatory mechanisms dictating the switch between the two respiratory quinones across the *Streptomyces* life cycle can provide novel insights into bacterial oxygen sensing and respiratory adaptations in the future.

Another noteworthy aspect of this study is the identification of a new freshwater *Streptomyces* strain, *Streptomyces* sp. MNU77. While commonly found in soil habitats, only 8 freshwater *Streptomyces* spp. have been discovered to date (27). Identification of *Streptomyces* species from unusual biogeographical niches such as deserts, highlands, and marine sediments represents a reservoir of untapped metabolic diversity. In contrast to soil-dwelling *Streptomyces* species, the bioactive potential of freshwater *Streptomyces* members remains relatively unexplored. Identification of *Streptomyces* sp. MNU77 thus provides a future opportunity to take advantage of the secondary metabolite discovery and production potential of freshwater *Streptomyces* spp.

## MATERIALS AND METHODS

### *Streptomyces* sp. MNU77 isolation and genome sequencing.

The 16S rRNA gene was predicted using the RNAmmer web server v.1.2. The predicted 16S rRNA gene sequence was compared with the GenBank database to identify the most similar sequences. The closest related strains were identified using EzBioCloud. The 16S rRNA gene sequences from the closest strains were pairwise aligned using ClustalW, and phylogenetic analysis was performed using the neighbor joining method in MEGA v.7.07.

### Culture conditions.

*Streptomyces* sp. MNU77 was routinely grown in tryptic soy broth at pH 9, 30°C, and 200 rpm for routine growth. Then, 1.5% agar was added for growth as solid plate colonies for 7 days to obtain *Streptomyces* sp. MNU77 spores. For standing liquid culture growth, 5% inoculum of *Streptomyces* sp. MNU77 culture was added to tryptic soy broth in a 35-mm 6-well plate. The plate was covered with parafilm and left undisturbed at 30°C. After 3 days, vegetative hyphae were harvested, and after 7 days, aerial hyphae were collected.

### Computational analysis.

The *Streptomyces* sp. MNU77 whole-genome shotgun sequence was downloaded from the NCBI nucleotide core database. The genome was run through antiSMASH v.5, PRISM, SBSPKS v.3, and RiPPMiner-Genome to search for natural product (PKS, NRPS, RiPP) BGCs. The CGView web server was used to plot the circular genome diagram. The core biosynthetic genes were color-coded according to the class of their product. The Simplified Molecular Input Line Entry System (SMILES) for the BGC products were retrieved from SBSPKSv3 and RiPPMiner-Genome output; these SMILES were used to construct the product structures in MarvinSketch.

### RNA isolation.

Total RNA from the desired cultures was isolated following the TRIzol method. Briefly, cultures were spun down and washed with 1× phosphate-buffered saline (PBS). Cells were homogenized by bead beating using 0.5-mm zirconia beads. The homogenate was centrifuged at 13,000 rpm for 5 min, and supernatant was collected. A one-fifth volume of chloroform was added and vortexed for 15 s. The mix was then incubated at room temperature for 15 min followed by centrifugation at maximum speed. The aqueous layer was then carefully collected in a fresh Eppendorf tube followed by isopropanol precipitation. After subsequent ethanol washes, RNA was air dried and resuspended in nuclease-free water. The samples were then run on 1.5% agarose gel to check for RNA integrity.

### cDNA synthesis and gene expression analysis.

Using the PrimeScript first-strand cDNA synthesis kit, cDNA was generated from 500 ng of total RNA from each specified sample. RT-qPCRs were prepared using 1 μL of cDNA reaction mixture for each gene-specific primer per reaction with SYBR green master mix following the manufacturer’s instructions in a Roche LightCycler 480 instrument II. Template normalization was performed by dividing the absolute gene expression of specific genes by the absolute gene expression of 16S rRNA. Reactions without the cDNA were used as a no-template negative control. Primer details are provided in Table S1.

### Metabolite extraction and analysis.

Cultures were harvested and suspended in an appropriate volume of 100 mM Tris-Cl (pH 8.0) and disrupted. The resultant whole-cell lysate was acidified by addition of 6 M HCl. Low-molecular-weight molecules were then extracted by adding a double volume of ethyl acetate and left overnight on a stirrer. The organic layer was then separated and evaporated to dryness, and the residual material was dissolved in a minimal volume of methanol and analyzed by information-dependent acquisition (IDA) scanning on a Sciex X500R quadrupole time of flight (QTOF) mass spectrometer fitted with an ExionLC UPHLC system using Sciex OS software with a previously reported method (45, 46). The LC separation was achieved on a Gemini 5U C_18_ column (Phenomenex; 5 μm, 50 by 4.6 mm) coupled to a Gemini guard column (Phenomenex; 4 by 3 mm, Phenomenex security cartridge). All metabolites, I to IX, were analyzed by iminodiacetic acid (IDA) scanning in both positive- and negative-ionization mode using an electrospray ionization (ESI) source with solvent systems, flow rates, and a solvent gradient described earlier (45, 46). The total scan time for both the MS1 and MS2 spectra was 2.5 s, and a collision energy (volts) of 5 was used. The declustering potential and ion source voltage were set at 100 and 5,500 V respectively.

### Generation of *Streptomyces* sp. MNU77 type III PKS expressing the M. smegmatis strain carrying Δ*pkqA*.

The *Streptomyces* sp. MNU77 type III PKS gene was PCR amplified with template from *Streptomyces* sp. MNU77 genomic DNA using Thermococcus kodakaraensis (KOD) polymerase from Toyobo on a Veriti thermal cycler. Amplification conditions for genes were initial denaturation, 94°C for 2 min; denaturation, 94°C for 15 sec; annealing, 62°C for 45 sec; and elongation, 68°C for 1 min. Amplified genes were further cloned in pST-Hi mycobacterial integrative vector using NdeI and HindIII restriction enzymes and T4 DNA ligase (47). The confirmed clone was then electroporated into M. smegmatis strain carrying Δ*pkqA* (9). Primer details are provided in Table S1.

Clones were confirmed for expression of *Streptomyces* sp. MNU77 type III PKS by probing the lysate with α-FLAG antibody (1:1,000) overnight at 4°C.

### Biofilm formation and quantitation with crystal violet staining.

Inoculum (1%) of WT, Δ*pkqA*, and Δ*pkqA*:*SspMNU77t3pks* M. smegmatis strains were inoculated in Sauton’s medium supplemented with 2% glucose. The plates were tightly covered with parafilm and incubated for 7 days at 37°C. After 7 days, the biofilm that formed was either harvested for metabolomics studies or was used for crystal violet staining. For crystal violet staining, the supernatant was discarded, and the plate was washed twice with 1× PBS. The plate was then kept inverted over filter paper for it to dry. The 0.1% crystal violet solution was then added to each well and incubated for 30 min. After 30 min, the crystal violet solution was discarded, and the plate was rinsed twice with 1× PBS and then left to dry for acquiring images. For measurement of absorbance, 1 mL of 90% ethanol was added to the resuspended stained biofilm, and absorbance was measured at 595 nm.

### Data availability.

The *Streptomyces* sp. strain MNU77 genome sequence has been deposited at GenBank under BioProject no. PRJNA267646.
