# Supplementary material for: Respiratory Quinone Switches from Menaquinone to Polyketide Quinone during the Development Cycle in Streptomyces sp. Strain MNU77
Source: Microbiol Spectr. 2022 Dec 12;11(1):e02597-22. doi: 10.1128/spectrum.02597-22 (PMC9927152; doi:10.1128/spectrum.02597-22)
Supplement: Supplemental file 1 — Supplemental material. Download spectrum.02597-22-s0001.pdf, PDF file, 0.4 MB [file spectrum.02597-22-s0001.pdf]

Supplementary figures and table

Supplementary Table 1: List of primers

Primers used for cloning-

Primer ID	Sequence
Type III pks FP	GGCATATGATGCGGCGACCGGGCGGGTCT
Type III pks RP	GGAAGCTTCCAGCGCAGCAGCACCAGTTC

Primers used for gene expression analysis-

Primer ID	Sequence
Type III pks FP	CCCCAAGGTGCTGGAGAC
Type III pksRP	GAGGACGAGGACAGGTTGC
Methyltransferase FP	GAGGGCACTATCCGACGAT
Methyltransferase RP	ATGACCCGGGTGTTCCAG
Oxidoreductase FP	CCTACGACCGGCATCTGG
Oxidoreductase RP	GAGGTGAGCAGGCGGTAAC
MqnA FP	GCAAGTTCTGCGCCTTCTAC
MqnA RP	CGTAGTACTCCACGCCGAAG
MqnB FP	CGTCCTCACCGTCTCCAC
MqnB RP	CTCCAGTACGGGCACCTC
MqnD FP	GCAAGGTCCGTAACAAGCTC
MqnD RP	CATCACCCAGAACACCTTGA

(a)

Features	Chromosome
Size(bp)	8,868,350
G+C content(%)	71.70%
N50	8,219,841
L50	1
DNA scaffolds	4
Coding sequences	7729
No. of RNAs	85
COG clusters	1681
KOG clusters	573
Pfam clusters	2335

(b)

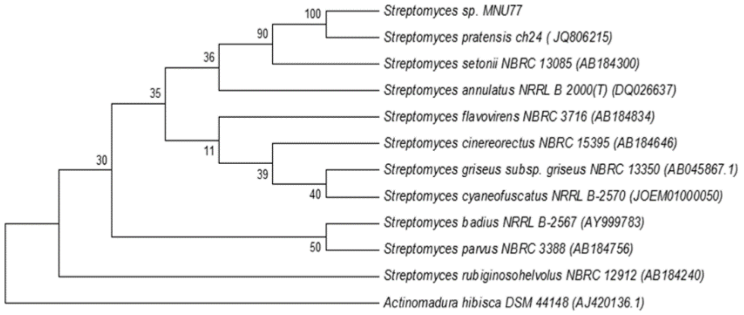

Figure S1 : Genomic and phylogenetic characterization of *Streptomyces sp. MNU 77*: (a) Genomic features of isolated *Streptomyces sp.* MNU77. **(b)** Phylogenetic analysis of *Streptomyces* strains using neighbour joining methods reveals *Streptomyces sp. MNU77* forms a distinct cluster with *Streptomyces pratensis*.

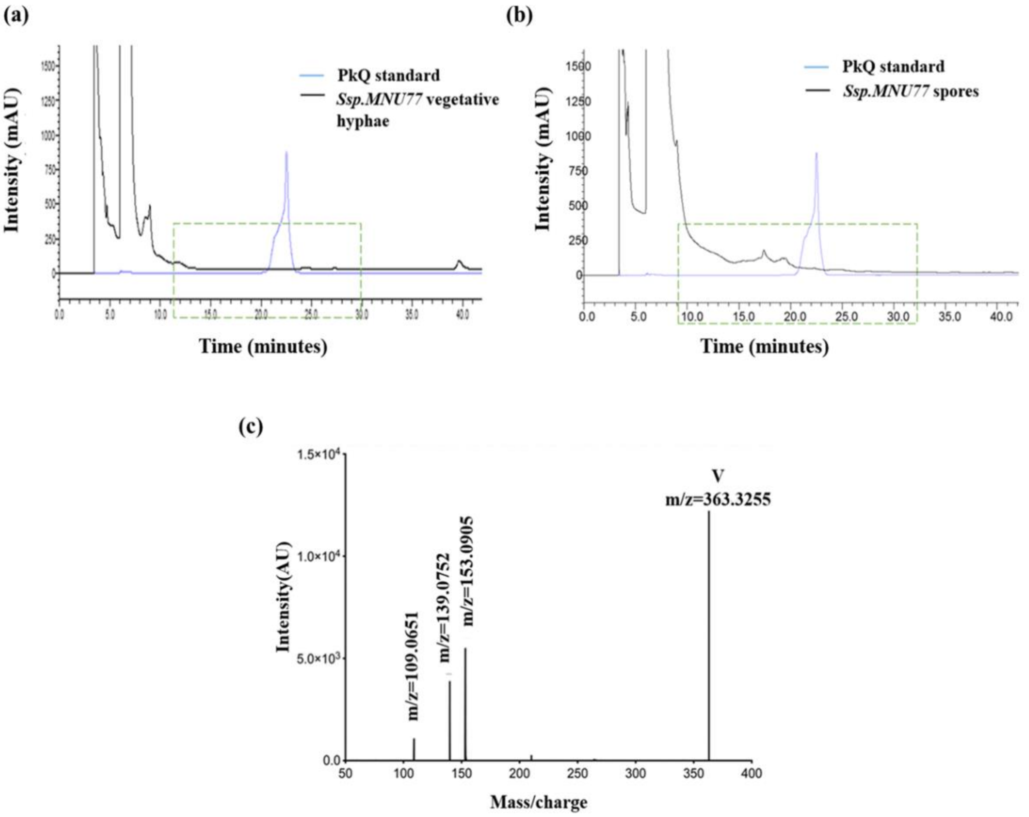

Figure S2: Identification and characterization of polyketide quinones from *Streptomyces sp. MNU77*: UPLC chromatogram of metabolite extract from **(a)** vegetative hyphaeand **(b)** spores of *Streptomyces sp. MNU77* overlaid synthetic PkQ standard. (**c**) MS/MS spectra of (**V**) m/z [M+H]+ = 363.32 detected from spores of *Streptomyces sp. MNU77*.
